# The HIF‐1α/p53/miRNA‐34a/Klotho axis in retinal pigment epithelial cells promotes subretinal fibrosis and exacerbates choroidal neovascularization

**DOI:** 10.1111/jcmm.16272

**Published:** 2021-01-12

**Authors:** Laiqing Xie, Ying Wang, Quan Li, Xiaoyan Ji, Yuanyuan Tu, Shu Du, Hui Lou, Xinwei Zeng, Linling Zhu, Ji Zhang, Manhui Zhu

**Affiliations:** ^1^ Department of Ophthalmology The Second Affiliated Hospital of Soochow University Suzhou China; ^2^ Department of Ophthalmology Suzhou Municipal Hospital The Affiliated Suzhou Hospital of Nanjing Medical University Suzhou China; ^3^ Department of Ophthalmology Lixiang Eye Hospital of Soochow University Suzhou China; ^4^ Center of Stomatology The Second Affiliated Hospital of Soochow University Suzhou China

**Keywords:** choroidal neovascularization, hypoxia‐inducible factor‐1α (HIF‐1α), Klotho, microRNA‐34a (miRNA‐34a), p53, subretinal fibrosis

## Abstract

Wet age‐related macular degeneration (wAMD), characterized by choroidal neovascularization (CNV), is a leading cause of irreversible vision loss among elderly people in developed nations. Subretinal fibrosis, mediated by epithelial‐mesenchymal transition (EMT) of retinal pigment epithelium (RPE) cells, leads to unsuccessful anti‐vascular endothelial growth factor (VEGF) agent treatments in CNV patients. Under hypoxic conditions, hypoxia‐inducible factor‐1α (HIF‐1α) increases the stability and activation of p53, which activates microRNA‐34a (miRNA‐34a) transcription to promote fibrosis. Additionally, Klotho is a target gene of miRNA‐34a that inhibits fibrosis. This study aimed to explore the role of the HIF‐1α/p53/miRNA‐34a/Klotho axis in subretinal fibrosis and CNV. Hypoxia‐induced HIF‐1α promoted p53 stability, phosphorylation and nuclear translocation in ARPE‐19 cells (a human RPE cell line). HIF‐1α‐dependent p53 activation up‐regulated miRNA‐34a expression in ARPE‐19 cells following hypoxia. Moreover, hypoxia‐induced p53‐dependent miRNA‐34a inhibited the expression of Klotho in ARPE‐19 cells. Additionally, the HIF‐1α/p53/miRNA‐34a/Klotho axis facilitated hypoxia‐induced EMT in ARPE‐19 cells. In vivo, blockade of the HIF‐1α/p53/miRNA‐34a/Klotho axis alleviated the formation of mouse laser‐induced CNV and subretinal fibrosis. In short, the HIF‐1α/p53/miRNA‐34a/Klotho axis in RPE cells promoted subretinal fibrosis, thus aggravating the formation of CNV.

## 1 INTRODUCTION

Age‐related macular degeneration (AMD) is the main cause of irreversible vision loss among elderly individuals in developed countries. Late AMD is categorized into two types, wet and dry, by the presence of choroidal neovascularization (CNV) or geographic atrophy (GA) involving the macular centre, respectively. Although wet AMD accounts for only 10% of AMD cases, this type is responsible for 90% of AMD‐induced vision loss.^1^ The reason for the high blindness ratio of wet AMD is that CNV may progress to end‐stage subretinal fibrosis. Subretinal fibrosis is characterized by complex interactions between cellular components and local inflammatory factors within subretinal lesions, which ultimately leads to the reconstruction of the extracellular matrix (ECM) and subretinal scar formation. Among these interactions, epithelial‐mesenchymal transition (EMT) of retinal pigment epithelium (RPE) cells is the critical contributor.^2^ In the context of AMD, RPE cells lose their cell‐cell adhesions and apical‐basal polarity, transforming into mesenchymal cells via EMT.^3^ Multiple extracellular ligands, such as galectin‐1^4^ and interleukin‐2 (IL‐2),^5^ are involved in the initiation and development of the EMT programme in RPE cells.^6^ Thoroughly studied for its central role in control of EMT, the ligand transforming growth factor‐beta (TGF‐β) is identified as the master regulator of EMT process.^7^, ^8^ At present, anti‐vascular endothelial growth factor (VEGF) agents have become the first‐line drugs for the treatment of CNV. Although anti‐VEGF agents usually stabilize or improve visual acuity, subretinal fibrosis develops in approximately 50% of treated eyes within 2 years following anti‐VEGF therapy.^9^ The formation of subretinal fibrosis can lead to local damage to the RPE, photoreceptors and choroidal vessels, resulting in continuous malfunction of the macular visual system. Thus, targeting subretinal fibrosis is a novel strategy for the treatment of CNV.

Until now, the pathogenesis of CNV is still vague. However, accumulating studies have revealed that hypoxia facilitates the progression of CNV. Hence, hypoxia‐inducible factor‐1α (HIF‐1α) is the main regulator of oxygen homeostasis that participates in wet AMD. HIF‐1α acts as a transcription factor of numerous target genes, among which p53 can promote fibrosis in the kidney^10^ and lung.^11^ P53 is also a transcription factor that is induced by hypoxia. HIF‐1α not only binds to the hypoxia response element 3 (HRE3) region on the p53 promoter to activate the transcription of p53,^12^ but also enhances the stability of p53^13^ and promotes the phosphorylation and nuclear translocation of p53.^14^ Therefore, we investigated whether HIF‐1α up‐regulates p53 expression, stability and activation under hypoxic conditions to promote subretinal fibrosis during CNV.

MicroRNA (miRNA) is a type of small non‐coding RNA that post‐transcriptionally modulates gene expression. MiRNA‐34a (miRNA‐34a) is a well‐known miRNA regulated by p53. Similar to p53, miRNA‐34a promotes lung^15^ and liver^16^ fibrosis. An Italian group determines that many miRNAs, including miRNA‐34a, are up‐regulated in the serum of neovascular AMD patients.^17^ Additionally, miRNA‐34a is significantly up‐regulated, while tolerance to oxidative stress is reduced, in hydrogen peroxide‐induced prematurely senescent ARPE‐19 cells (a human RPE cell line),^18^ indicating that miRNA‐34a is involved in CNV.

Klotho (KL) is a membrane‐bound protein that plays an anti‐ageing role because *Kl*‐null mice exhibit phenotypes similar to human premature ageing syndromes.^19^ The level of circulating Klotho decreases with age and thereby increases the risk for age‐associated diseases.^20^ In cultured human RPE, KL up‐regulates the expression of stress‐related genes and reduces the production of reactive oxygen species (ROS), thus protecting RPE from oxidative stress‐induced injury,^21^ suggesting that KL plays a protective role in CNV. MiRNA‐34a down‐regulates Klotho protein levels by directly binding to the three‐prime untranslated region (3' UTR) of KL. Moreover, miRNA‐34a promotes renal fibrosis by down‐regulating KL in tubular epithelial cells.^22^

Herein, we sought to determine the role of the HIF‐1α/p53/miRNA‐34a/Klotho axis in subretinal fibrosis in CNV. Our data provide a novel approach for the treatment of wet AMD.

## 2 MATERIALS AND METHODS

### 2.1 Cell culture

The human RPE cell line ARPE‐19 (#CRL‐2302, American Type Culture Collection) was cultured in DMEM containing glutamine and supplemented with 50 U penicillin/50 mg streptomycin and 10% foetal bovine serum (FBS) (#F8687, Sigma‐Aldrich). ARPE‐19 cells in the normoxia (normal) group were cultured under 95% O_2_/5% CO_2_, and ARPE‐19 cells in the hypoxia group were cultured under 95% N_2_/5% CO_2_ in an incubator (Model 3130; Thermo Fisher Scientific).

### 2.2 **Cycloheximide (CHX) chase assa**y

ARPE‐19 cells cultured under normoxia (normal), hypoxia, hypoxia + 0.1% DMSO (the same volume as that of digoxin for 24 hours) or hoursypoxia + digoxin (HIF‐1α inhibitor; #S4290, Selleckchem; diluted in 0.1% DMSO; 0.5 μmol/L for 24 hours) were mixed with 10 μg/mL CHX, and then, p53 protein levels were measured by Western blot at 0, 15, 30 and 60 minutes.

### 2.3 Western blot

Total proteins derived from ARPE‐19 cells or mouse retina‐RPE‐choroid tissues were extracted by lysis with ice‐cold RIPA buffer (#ab156034, Abcam) containing 1% (volume/volume) protease and phosphatase inhibitor cocktail (#PPC1010, Sigma‐Aldrich). The extracted proteins (80 µg in each lane) were separated by 10% SDS‐PAGE (#NP0326BOX, Thermo Fisher Scientific) and then transferred to PVDF membranes (#88518, Thermo Fisher Scientific). Next, the membranes were incubated with primary and secondary antibodies. Primary antibodies included anti‐HIF‐1α (#ab82832, Abcam), anti‐p‐p53 (S15; #ab1431, Abcam), anti‐p‐p53 (S20; #PA5‐104741, Thermo Fisher Scientific), anti‐p‐p53 (S46; #SAB4504503, Merck), anti‐p53 (#MA1‐12549, Thermo Fisher Scientific), anti‐Klotho (#NBP1‐76511, Novus Biologicals), anti‐fibronectin (Fib) (#ab2413, Abcam), anti‐N‐cadherin (N‐cad) (#ab76057, Abcam), anti‐vimentin (Vim) (#ab137321, Abcam), anti‐GAPDH (#G9545, Sigma‐Aldrich) and anti‐histone 3 (H3) (#ab1791, Abcam). Secondary antibodies included horseradish peroxidase (HRP)‐conjugated goat anti‐rabbit IgG (12‐34) and HRP‐conjugated goat anti‐mouse IgG (#A5278) (both were purchased from Sigma‐Aldrich). The blots were developed by enhanced chemiluminescence.

### 2.4 Isolation of nuclear and cytoplasmic proteins

Nuclear and cytoplasmic proteins of ARPE‐19 cells were isolated via NE‐PER^®^ nuclear and cytoplasmic extraction reagents (#78833, Thermo Fisher Scientific).

### 2.5 Quantitative real‐time PCR (qRT‐PCR)

Quantitative real‐time PCR was performed as previously described.^23^ U6 small nuclear RNA (snoRNA) was used as the internal control for miRNA‐34a and pri‐miRNA‐34a. GAPDH mRNA was used as the internal control for Klotho. The relative levels of miRNA‐34a, pri‐miRNA‐34a and Klotho were calculated using the 2^‐ΔΔCt^ method. The primers were as follows: miRNA‐34a (forward 5′‐GCCCTGGCAGTGTCTTAG‐3′ and reverse 5′‐CAGTGCGTGTCGTGGAGT‐3′), pri‐miRNA‐34a (forward 5′‐CCAGCTGTGAGTGTTTCTTTGGCAG‐3′ and reverse 5′‐CCCACAACGTGCAGCACTTCTAG‐3′), U6 snoRNA (forward 5′‐CGCTTCGGCAGCACATATACTAA‐3′ and reverse 5′‐TATGGAACGCTTCACGAATTTGC‐3′), human Klotho (forward 5′‐TAGCCAGCGACAGCTACAAC‐3′ and reverse 5’‐GAAGCGGTAGTGAGTGACCC‐3’) and GAPDH (forward 5′‐GACAGTCAGCCGCATCTTCT‐3′ and reverse 5′‐GCGCCCAATACGACCAAATC‐3′).

### 2.6 Dual‐luciferase reporter assay

Adeno‐associated virus (AAV)‐p53 (#AAV‐226227) and AAV‐p53 mutant (S15A, S20A and S46A) were purchased from Vector Biolabs. The wild‐type miRNA‐34a promoter region (34a WT) and mutant miRNA‐34a promoter regions (34a MUT1, 34a MUT2, 34a MUT3 and 34s MUT1/2/3) were amplified from ARPE‐19 cell genomic DNA and cloned into the pGL3 basic luciferase vector (#E1751, Promega). Fragments of the human Klotho 3’ UTR containing putative‐binding sites were amplified by PCR using human genomic DNA as a template. The PCR products were inserted into the p‐MIR‐REPORT plasmid (#AM5795, Invitrogen) and verified by sequencing. The primers for human 3′ UTR‐Klotho were 5′‐GCCGAGCTCATGGGCATAGGTGATCGTAA‐3′ and 5′‐CGCAAGCTTTCCACAGAAGTAGCAGCAAA‐3′. To verify the binding specificity, the putative‐binding sites of miRNA‐34a in the Klotho 3′ UTR were mutated by PCR (from 5′‐CUGCCA‐3′ to 5′‐GUTGCA‐3′, as shown in Figure 3B).

In the luciferase reporter assay, ARPE‐19 cells were cultured in 24‐well plates, and each well was co‐transfected with firefly luciferase reporter plasmid (500 ng) and with miRNA‐34a mimic or negative control RNA with Lipofectamine 3000 (#L3000008, Thermo Fisher Scientific). An internal control reporter plasmid (10 ng) expressing *Renilla reniformis* luciferase was co‐transfected to normalize the transfection efficiency. Luciferase activities were measured 24 hours after transfection using a Renilla luciferase assay system (#E2810, Promega). Relative luciferase activity (arbitrary units) was expressed as fold changes over the control group after normalizing for the transfection efficiency.

### 2.7 Chromatin immunoprecipitation (ChIP)

Four micrograms of DO‐1 anti‐p53 monoclonal antibody (#ab1101, Abcam) was used. Isotype‐matched pre‐immune mouse IgG (#ab188776, Abcam) was used as a negative control. Quantitative real‐time PCR with EvaGreen dye technology (Bio‐Rad) was used for the analysis of the DNA in ChIP samples. ChIP data analysis was performed using the fold enrichment method and normalizing to the mock (IgG) control for each sample. The oligonucleotides used for positive and negative controls were as previously described [59]. The p21 promoter (5′ p53 response element) was amplified with the primers forward, 5′‐CTGGACTGGGCACTCTTGTC‐3′ and reverse 5′‐CTCCTACCATCCCCTTCCTC‐3′. The negative control promoter was amplified with the primers forward, 5′‐GGAGTCCTGTTTGCTTCTGG‐3′ and reverse 5′‐CTTTGGCCACACTGAGGAAT‐3′ and was used as previously described.^24^

### 2.8 Mouse CNV model construction

Male C57BL/6J mice aged 6‐9 weeks (Laboratory Animal Center of Soochow University, China) were used in the study. All animal experimental procedures were approved by the Committee on the Ethics of Animal Experiments of Soochow University. Laser photocoagulation of the retina was performed to induce CNV lesions as previous description.^25^ The mice were divided into the following groups: normal, CNV 7 days, CNV 7 days + 0.1% DMSO, CNV 7 days + digoxin (oral; 2 mg/kg for 7 days), CNV 7 days + AAV‐p53 mutant (intravitreal injection; approximately 3 μL, 3 × 10^10^ viral particles/mL), CNV 7 d + miRNA‐34a inhibitor (intravitreal injection; 1 μg) and CNV 7 days + AAV‐Klotho full‐length plasmid (intravitreal injection, 2 μL, 5 × 10^10^ viral particles/mL). The CNV model was constructed on day 1. The intravitreal injections were performed on day 4. The mice were killed on day 7. For fundus angiography (FFA and ICGA) and fluorescent staining of choroidal flat mounts, there were four mice in each six group, totally 72 mice. For Western blot, there were five mice in each seven group, totally 35 mice. Besides there were four mice excluded from the experiments due to haemorrhage on the photocoagulation site.

### 2.9 Fundus angiography

Fundus fluorescein angiography (FFA) and indocyanine green angiography (ICGA) were performed 7 days after CNV. The mice were anaesthetized, their pupils were dilated, and then, they were intraperitoneally injected with 5 μg/g fluorescein AK‐FLUOR (#17478025310, Akorn) or 0.075 μg/g indocyanine green (ICG) (#1340009, Sigma‐Aldrich). Fundus fluorescent images were obtained using a retinal imaging microscope (Micron IV, Phoenix Research Laboratories) 5 minutes and 10 minutes after fluorescein injection. In addition, fluorescein leakage was graded by two independent blinded observers using previously established criteria.^26^ The total CNV area was analysed from ICGA images using ImageJ software.

### 2.10 Fluorescent staining of choroidal flat mounts

On d 7 after CNV, after the removal of the anterior portion, radial relaxation incision was made in the eyecup of the sclera/choroid/RPE complexes. Then immunostaining was performed in choroidal flat mounts. The choroidal tissues were incubated with a 1 mg/mL solution of Alexa Fluor 488‐conjugated IB4 (#I21411, Thermo Fisher Scientific) and Alexa Fluor 594‐conjugated collagen type I (#bs‐10423R‐A594, Bioss Inc) antibodies at 4°C for 24 hours.

### 2.11 Statistical analyses

Experimental results are represented as the mean ± SEM. All data were analysed by Student's *t* test or one‐way ANOVA with Tukey's post hoc test via GraphPad Prism software. A *P* value <.05 indicated significance.

## 3 RESULTS

### 3.1 Hypoxia‐induced HIF‐1α promotes p53 stability, phosphorylation and nuclear translocation in ARPE‐19 cells

**FIGURE 1:**
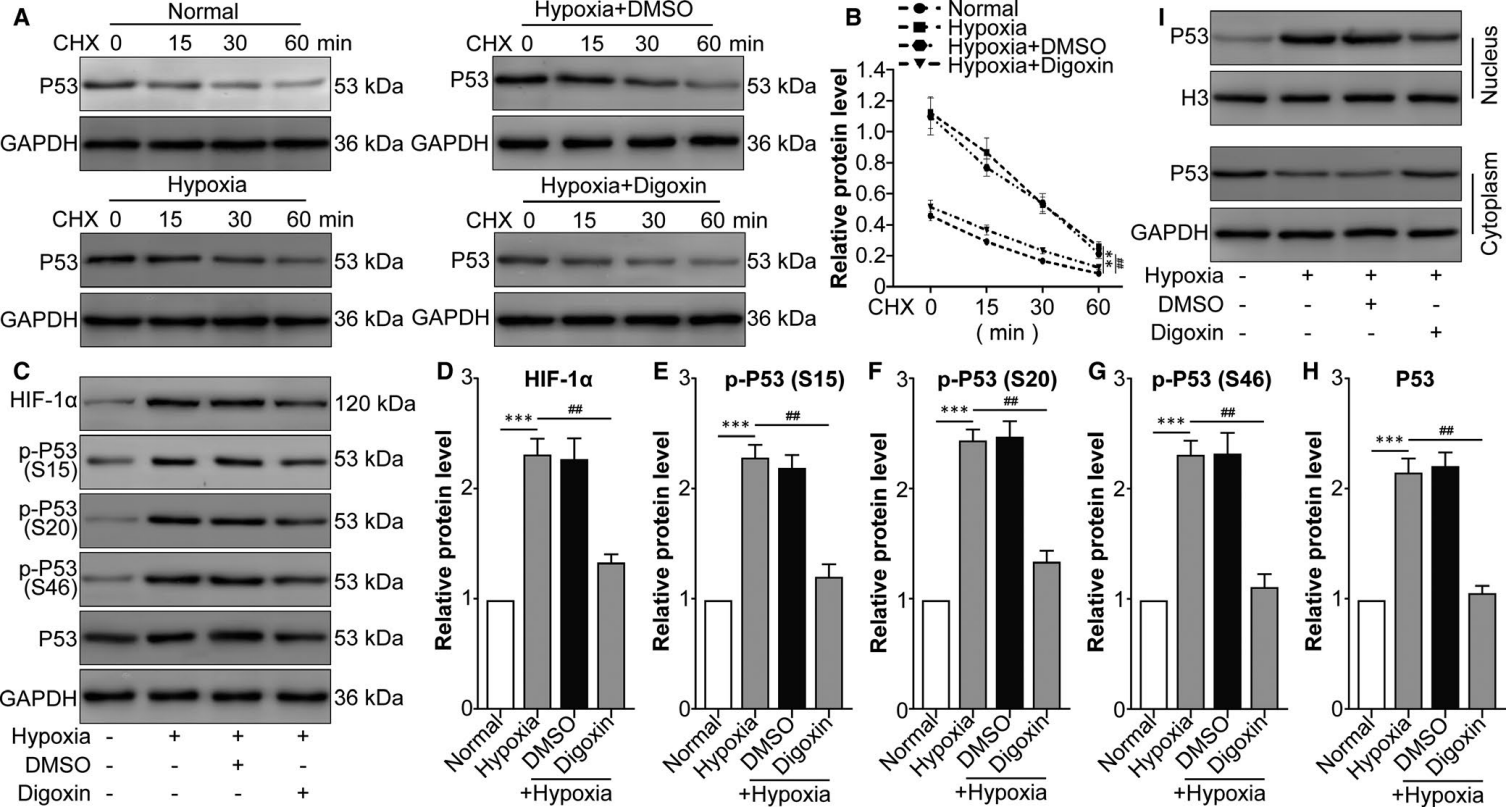
Hypoxia‐induced HIF‐1α increases p53 stabilization, phosphorylation and nuclear translocation in ARPE cells. ARPE‐19 cells were divided into the following groups: normal, hypoxia, hypoxia + 0.1% DMSO (the same volume as digoxin for 24 h) and hypoxia + digoxin (HIF‐1α inhibitor; 0.5 μmol/L for 24 h). A, After different treatments, CHX (100 μg/mL) was used to treat human RPE cells prior to Western blot analysis. B, ImageJ was used to quantify p53 band densitometry. ^**^ *P* < .01, hypoxia group vs normal group. ^##^ *P* < .05, hypoxia + digoxin group vs hypoxia group. C, Western blot was performed to measure HIF‐1α, p‐p53 (S15), p‐p53 (S20), p‐p53 (S46) and p53 protein levels. The relative protein level of HIF‐1α compared with the GAPDH level (D) and the ratio of p‐p53 (S15)/p53 (E), p‐p53 (S20)/p53 (F), p‐p53 (S46)/p53 (G) and p53/GAPDH (H) were analysed. ^***^ *P* < .001, hypoxia group vs normal group. ^##^ *P* < .01, hypoxia + digoxin group vs hypoxia group. I, Nuclear and cytoplasmic separation samples were prepared, and Western blot was performed to measure p53 protein levels. Histone 3 (H3) and GAPDH were used as the nuclear and cytoplasmic markers, respectively

First, p53 stabilization under hypoxic conditions regulated by HIF‐1α was detected by CHX treatment, which showed that compared with the normal treatment, hypoxia increased p53 protein stability, while the HIF‐1α inhibitor digoxin decreased p53 protein stability (Figure 1A,B). The activation of p53 is dependent on its phosphorylation at S15, S20 and S46 sites.^27^ Western blot showed that HIF‐1α, p‐p53 (S15), p‐p53 (S20) and p‐p53 (S46) increased following hypoxia compared with their levels in the normal group, while digoxin down‐regulated HIF‐1α expression and phosphorylation of p53 (Figure 1C‐H). As a transcription factor, p53 enters the nucleus after its activation.^28^ Nuclear and cytoplasmic separation showed that hypoxia induced the nuclear translocation of p53, which was inhibited by digoxin (Figure 1I). Collectively, the above data indicated that hypoxia‐induced HIF‐1α promoted p53 stability, phosphorylation and nuclear translocation in ARPE‐19 cells.

### 3.2 HIF‐1α–dependent p53 activation up‐regulates miRNA‐34a expression in ARPE‐19 cells after hypoxia

**FIGURE 2:**
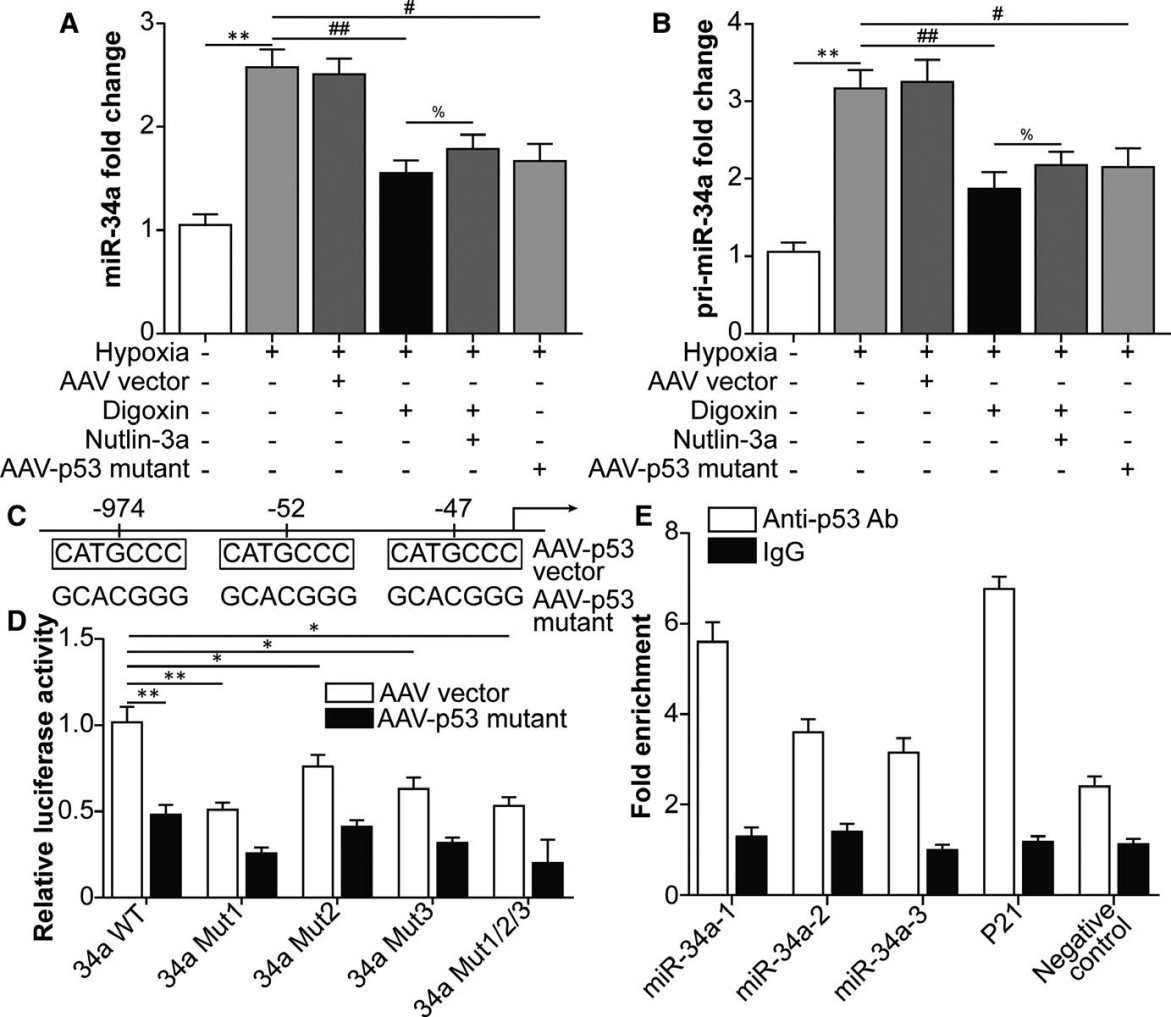
Hypoxia‐inducible factor‐1α –dependent p53 activation promotes miRNA‐34a expression in ARPE‐19 cells following hypoxia. Human RPE cells were divided into the following groups: normal, hypoxia, hypoxia + AAV vector, hypoxia + digoxin, hypoxia + digoxin + nutlin‐3a (MDM2 inhibitor and p53 agonist; 5 μmol/L for 24 h) and hypoxia + AAV‐p53 mutant (S15A, S20A and S46A) infection. A, qRT‐PCR was performed to measure miRNA‐34a levels in ARPE‐19 cells. B, qRT‐PCR was performed to measure pri‐miRNA‐34a levels in ARPE‐19 cells. In Figure 2A,B, ^**^ *P* < .01, hypoxia group vs normal group. ^##^ *P* < .01, ^#^ *P* < .05, compared with the hypoxia group. ^%^ *P* < .05, hypoxia + digoxin + nutlin‐3a group vs hypoxia + digoxin group. C, The p53‐binding sites in the miRNA‐34 promoter. D, P53 regulated the miRNA‐34a promoter. The p53 mutant resulted in a decrease in miRNA‐34a promoter activity (miRNA‐34a WT). The mutagenesis of the three p53‐binding sites, singularly (miRNA‐34a mutant1, miRNA‐34a mutant2 and miRNA‐34a mutant3) or in combination (miRNA‐34a mutant1/2/3), abrogated this effect. E, ChIP was conducted with an anti‐p53 antibody on ARPE‐19 genomic DNA. The immunoprecipitated chromatin was found to be enriched with the target miRNA‐34a promoter (miRNA‐34a‐1, miRNA‐34a‐2 and miRNA‐34a‐3, the regions encompassing each of three p53‐binding sites) by qPCR. Data are reported as fold enrichment over control samples (immunoprecipitation with IgG)

A previous study revealed that p53 induces microRNA‐34a (miRNA‐34a) expression in multiple cancer cells, including osteosarcoma and breast cancer cells, as well as in irradiated mice, by binding to a specific p53‐binding site in the gene that encodes miRNA‐34a.^29^ Therefore, miRNA‐34a in ARPE‐19 cells was detected, which showed that HIF‐1α–dependent p53 activation promotes miRNA‐34a expression (Figure 2A). The discovery that modification of p53 affected miRNA‐34a expression at the transcript level (pri‐miRNA; Figure 2B) further supported the speculation that p53 enhanced miRNA‐34a transcription in ARPE‐19 cells. As shown in Figure 2C, three CATGCCC sequences at the sites −974, −52 and −47 in the miRNA‐34a promoter were predicted as p53‐binding sites. We then generated a miRNA‐34a reporter plasmid containing the miRNA‐34a promoter, either WT or mutant of three p53‐binding sites (p53BS, miRNA‐34a‐1, miRNA‐34a‐2 and miRNA‐34a‐3), which was cloned upstream of the luciferase gene. Single p53BS mutant (34aMUT1, 34aMUT2 and 34aMUT3) and p53BS triple site mutant (34aMUT1/2/3) reporters were generated. The reporter assays showed that the p53 mutant had little effect on the reporters in which either one or all three p53BS were mutated (Figure 2D white vs black matched columns). In addition, the luciferase activity of p53‐overexpressing cells was reduced by the p53BS mutant reporters compared with WT reporter (Figure 2D comparison between white columns). This difference was abolished by p53 mutant infection (Figure 2D comparison between black columns). These data suggest that miRNA‐34a is a direct transcriptional target of p53. To further confirm that p53 bound to the miRNA‐34a promoter, we performed ChIP. ARPE‐19 cell lysates were immunoprecipitated with a p53 antibody, and the regions surrounding the three p53BS elements in the miRNA‐34a promoter (miRNA‐34a‐1, miRNA‐34a‐2 and miRNA‐34a‐3) were amplified and quantified by quantitative PCR (qPCR). The qPCR‐ChIP assay verified that the amplicons surrounding three p53BS elements were highly enriched compared with those in the negative control (Figure 2E). The evaluation of p53 functional binding sites in the miRNA‐34a promoter region strongly demonstrated that miRNA‐34a is a direct transcriptional target of p53 in ARPE‐19 cells.

### 3.3 Hypoxia‐induced p53‐dependent miRNA‐34a inhibits the expression of Klotho in ARPE‐19 cells

**FIGURE 3:**
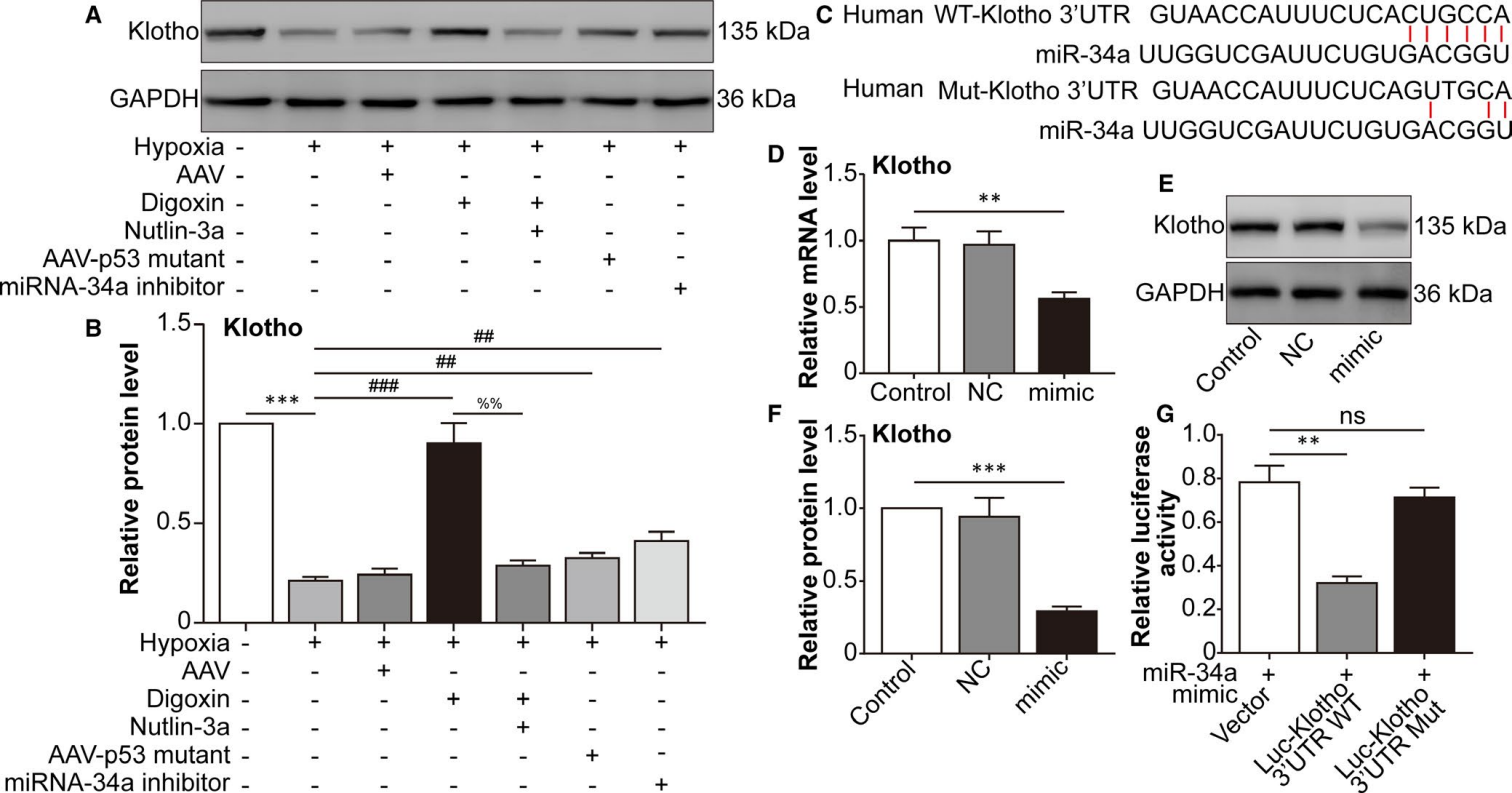
microrna‐34a inhibits the expression of Klotho. ARPE‐19 cells were divided into the following groups: normal, hypoxia, hypoxia + AAV vector, hypoxia + digoxin, hypoxia + digoxin + nutlin‐3a, hypoxia + AAV‐p53 mutant infection and hypoxia + miRNA‐34a inhibitor. A, Western blot assay of Klotho in ARPE‐19 cells was performed. B, The relative protein level of Klotho compared with the GAPDH level was analysed. ^***^ *P* < .001, hypoxia group vs normal group. ^###^ *P* < .001, ^##^ *P* < .01, compared with the hypoxia group. ^%%^ *P* < .01, hypoxia + digoxin + nutlin‐3a group vs hypoxia + digoxin group. C, Schematic of the Klotho 3' UTR with the WT or mutated putative‐binding site of miRNA‐34a inserted into a luciferase (Luc) reporter. D, qRT‐PCR analysis of Klotho mRNA expression in HEK293T cells transfected with miRNA‐34a mimic for 24 h. ^**^ *P* < .01, miRNA‐34a mimic group vs control group. E, Western blot of Klotho in HEK293T cells transfected with a miRNA‐34a mimic for 24 h was performed. F, Immunoblotted Klotho was quantified and normalized to GAPDH. ^***^ *P* < .001, miRNA‐34a mimic group vs control group. G Relative luciferase activity was detected in HEK293T cells co‐transfected with plasmids containing firefly luciferase and wild‐type (WT) or mutant (Mut) Klotho 3′‐UTR and miRNA‐34a mimic for 24 h. The luciferase activity values were normalized to *Renilla reniformis* luciferase (TK‐RL) activity. ^**^ *P* < .01, Luc‐Klotho 3′ UTR WT group vs vector group. Statistically non‐significant (NS), Luc‐Klotho 3′ UTR mutant group vs vector group

Next, we aimed to identify the target mRNA of miRNA‐34a. Overexpression of miRNA‐34a promotes epithelial‐to‐mesenchymal transition (EMT) in human renal tubular epithelial HK‐2 cells, together with down‐regulation of Klotho, which is an endogenous antagonist of renal fibrosis. Furthermore, a luciferase reporter assay revealed that miRNA‐34a down‐regulated Klotho expression by directly binding to the 3′ UTR of Klotho mRNA.^22^ Thus, we speculated that miRNA‐34a inhibited Klotho expression in hypoxia‐challenged ARPE‐19 cells. Western blot analysis showed that hypoxia‐down‐regulated Klotho protein levels were reversed by HIF‐1α inhibition, p53 mutation and miRNA‐34a inhibition. P53 activation increased the effect of HIF‐1α inhibition (comparison between hypoxia + digoxin and hypoxia + digoxin +nutlin‐3a groups), indicating that HIF‐1α functioned via downstream p53 (Figure 3A,B). As shown in Figure 3C, miRNA‐34a bound to the 3′ UTR of Klotho mRNA. As expected, in ARPE‐19 cells, Klotho decreased at both the mRNA and protein levels after miRNA‐34a mimic transfection (Figure 3D‐F). To determine whether miRNA‐34a directly affected Klotho mRNA, a luciferase reporter that contained a WT or mutated binding site of miRNA‐34a in the 3’ UTR of the Klotho gene was generated and then transfected into ARPE‐19 cells. Interestingly, the transfection of the miRNA‐34a mimic inhibited the luciferase activity of the WT‐Klotho reporter, but had a negligible effect on the activity of the mutant‐Klotho reporter (Figure 3G). These data indicated that miRNA‐34a down‐regulated Klotho expression by directly binding to the 3′ UTR of Klotho mRNA.

### 3.4 The HIF‐1α/p53/miRNA‐34a/Klotho axis facilitates hypoxia‐induced epithelial‐mesenchymal transition (EMT) in ARPE‐19 cells

**FIGURE 4:**
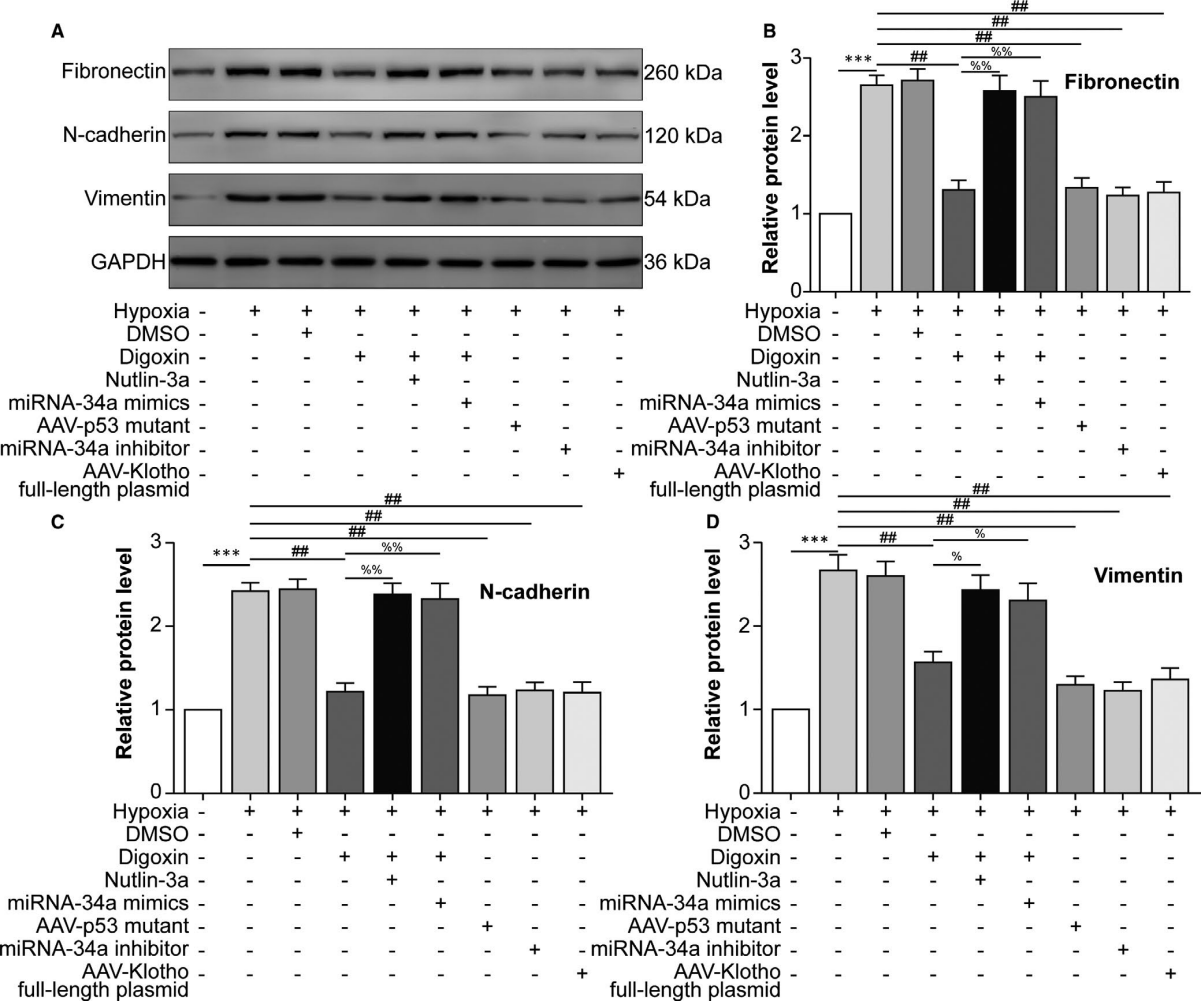
The HIF‐1α/p53/miRNA‐34a/Klotho axis facilitates hypoxia‐induced epithelial‐mesenchymal transition (EMT) in ARPE‐19 cells. ARPE‐19 cells were divided into the following groups: negative control (NC), hypoxia, hypoxia + 0.1% DMSO, hypoxia + digoxin, hypoxia + digoxin +nutlin‐3a, hypoxia + digoxin + miRNA‐34a mimics, hypoxia + AAV‐p53 mutant infection, hypoxia + miRNA‐34a inhibitor transfection (40 nM for 24 h) and hypoxia + AAV‐Klotho full‐length plasmid infection. A, Western blot was performed to measure the protein levels of the mesenchymal cell markers fibronectin (Fib), N‐cadherin (N‐cad) and vimentin (Vim). The relative protein levels of Fib (B), N‐cad (C) and Vim (D) compared with GAPDH levels were analysed. ^***^ *P* < .001, hypoxia group vs normal group. ^##^ *P* < .01, compared with the hypoxia group. ^%%^ *P* < .01, ^%^ *P* < .05, compared with the hypoxia + digoxin group

Our data suggested that hypoxia induced HIF‐1α expression to up‐regulate p53, followed by p53‐induced transcription of miRNA‐34a, which inhibited Klotho expression. Then, we examined whether the HIF‐1α/p53/miRNA‐34a/Klotho axis participated in EMT of ARPE‐19 cells following hypoxia treatment. Western blot showed that mesenchymal cell markers, including Fib, N‐cad and Vim, were induced by hypoxia, while HIF‐1α inhibition, p53 mutation, miRNA‐34a inhibition and Klotho overexpression (KL OE) via AAV‐Klotho full‐length plasmid infection decreased these markers. P53 activation, miRNA‐34a mimics and KL OE impaired the effect of HIF‐1α inhibition, indicating that HIF‐1α promoted EMT of ARPE‐19 cells via downstream up‐regulation of p53 and miRNA‐34a and down‐regulation of Klotho (Figure 4A‐D). These data suggested that the HIF‐1α/p53/miRNA‐34a/Klotho axis facilitated hypoxia‐induced EMT in ARPE‐19 cells.

### 3.5 Blockade of the HIF‐1α/p53/miRNA‐34a/Klotho axis mitigates mouse laser‐induced CNV lesions

**FIGURE 5:**
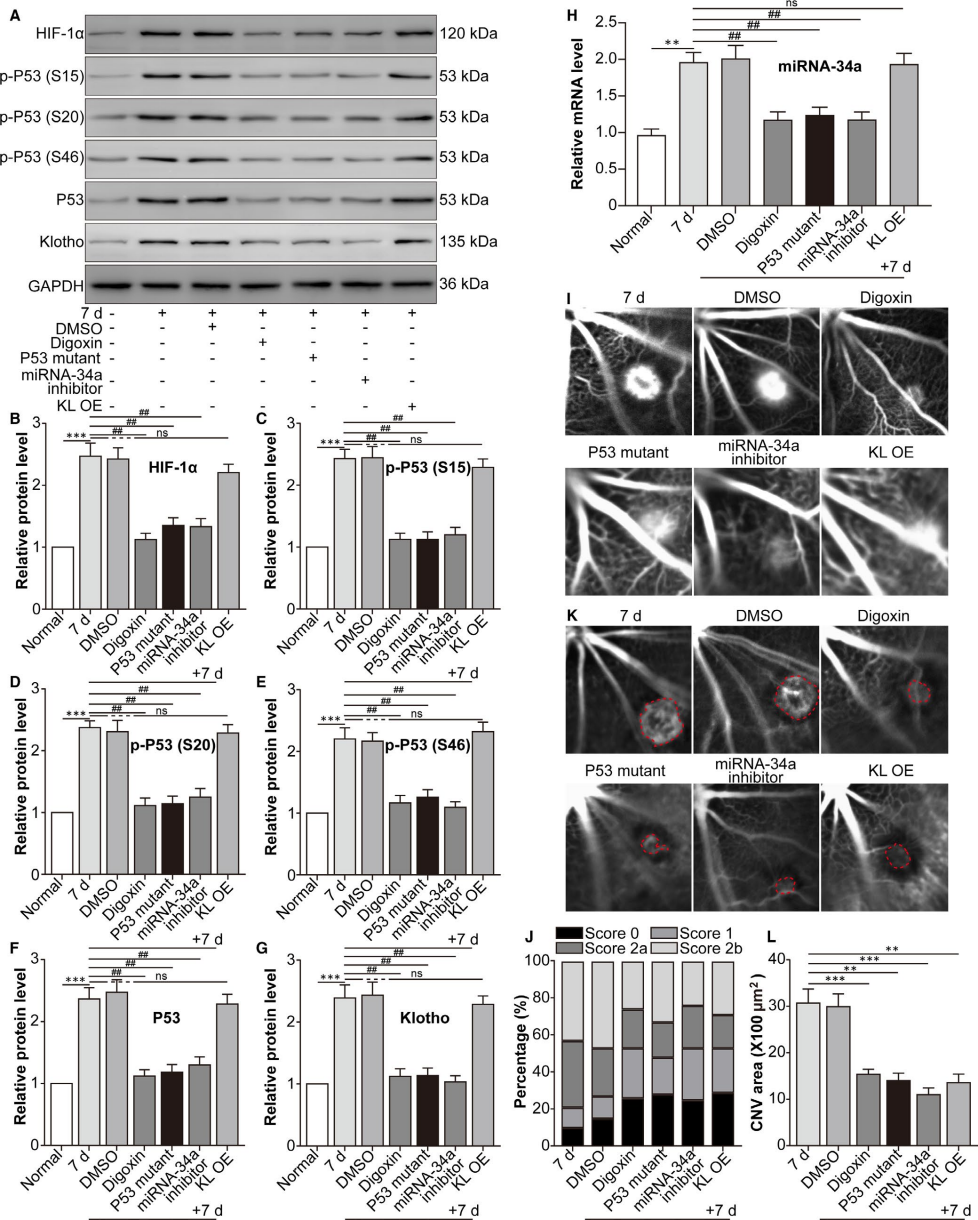
Blockade of the HIF‐1α/p53/miRNA‐34a/Klotho axis decreases the leakage and area of mouse laser‐induced CNV. The mice were divided into the following groups: normal, CNV 7 d, CNV 7 d + 0.1% DMSO, CNV 7 d + digoxin (oral; 2 mg/kg for 7 d), CNV 7 d + AAV‐p53 mutant (intravitreal injection; approximately 3 μL, 3 × 10^10^ viral particles/mL), CNV 7 d + miRNA‐34a inhibitor (intravitreal injection; 1 μg) and CNV 7 d + AAV‐Klotho full‐length plasmid (intravitreal injection, 2 μL, 5 × 10^10^ viral particles/mL). A, Western blot was performed to measure HIF‐1α, p‐p53 (S15), p53 (S20), p‐p53 (S46), p53 and Klotho protein levels. B, The relative protein levels of HIF‐1α/GAPDH (B), p‐p53 (S15)/p53 (C), p‐p53 (S20)/p53 (D), p‐p53 (S46)/p53 (E), p53/GAPDH (F) and Klotho/GAPDH (G) were analysed. ^***^ *P* < .001, CNV 7‐d group vs normal group. ^##^ *P* < .01, compared with the CNV 7‐d group. NS, CNV 7 d + KL OE group vs CNV 7‐d group. H, RT‐PCR was performed to measure the expression of miRNA‐34a. ^**^ *P* < .01, CNV 7‐d group vs normal group. ^##^ *P* < .01, compared with the CNV 7‐d group. NS, CNV 7 d + KL OE group vs CNV 7‐d group. I, FFA was performed to measure the leakage of CNV. J, The leakage of CNV was analysed. K, ICGA was performed to measure the area of CNV. L, The area of CNV was analysed. ^**^ *P* < .01, ^***^ *P* < .001, compared with the CNV 7‐d group

The EMT process of RPE cells contributes to CNV.^30^ Subsequently, we investigated the effect of the HIF‐1α/p53/miRNA‐34a/Klotho axis on mouse laser‐induced CNV. CNV‐induced HIF‐1α and phosphorylation of p53 and miRNA‐34a were reversed by HIF‐1α suppression, p53 mutation, miRNA‐34a inhibition and KL OE, while Klotho displayed the opposite tendency (Figure 5A‐H). Moreover, HIF‐1α suppression, p53 mutation, miRNA‐34a knockdown and KL OE alleviated CNV leakage (Figure 5I,J). Similarly, HIF‐1α suppression, p53 mutation, miRNA‐34a inhibition and KL OE also reduced the area (Figure 5K,L) of CNV. The data suggested that blockade of the HIF‐1α/p53/miRNA‐34a/Klotho axis alleviated leakage and the area of mouse laser‐induced CNV.

### 3.6 Blockade of the HIF‐1α/p53/miRNA‐34a axis mitigates subretinal fibrosis and decreases mouse laser‐induced CNV volume

**FIGURE 6:**
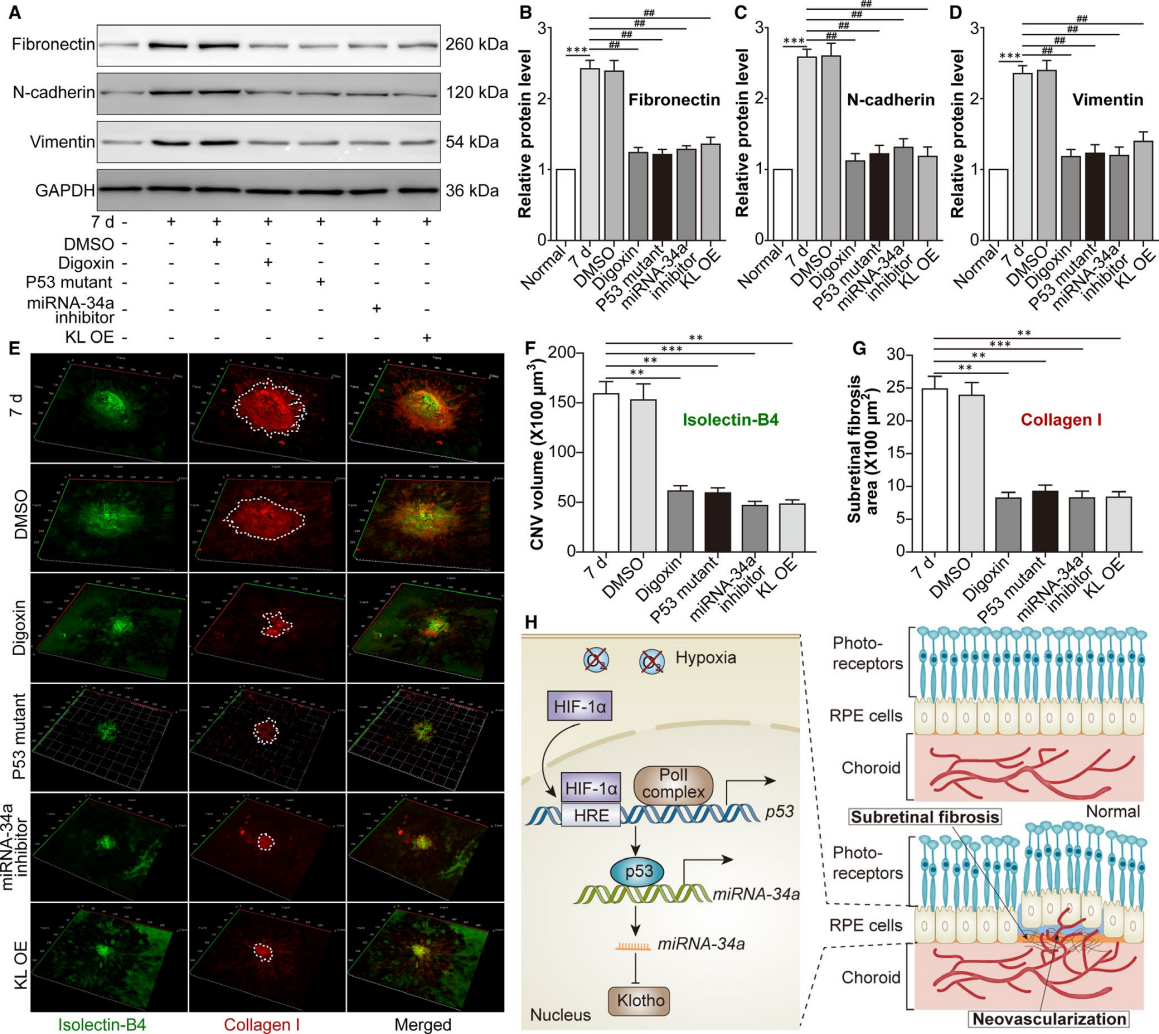
Blockade of the HIF‐1α/p53/miRNA‐34a axis mitigates subretinal fibrosis in a mouse CNV‐induced CNV model. The mice were divided into the following groups: normal, CNV 7 d, CNV 7 d + 0.1% DMSO, CNV 7 d + digoxin, CNV 7 d + AAV‐p53 mutant, CNV 7 d + miRNA 34a inhibitor and CNV 7 d + AAV‐Klotho full‐length plasmid. A, Western blot was performed to measure Fib, N‐cad and Vim protein levels in retina‐RPE‐choroid tissues. ^***^ *P* < .001, CNV 7‐d group vs normal group. ^##^ *P* < .01, compared with the CNV 7‐d group. B, Relative protein levels of Fib (B), N‐cad (C) and Vim (D) compared with GAPDH levels were analysed. E, IB4 (green) and type I collagen (colI; red) were stained on the choroidal flat mount. F, The volume of CNV was analysed. G, The subretinal fibrosis area was analysed. ^**^ *P* < .01, ^***^ *P* < .001, compared with the CNV 7‐d group in Figure 6F,G. H, A schematic diagram displaying the role of HIF‐1α/p53/miRNA‐34a in CNV is shown. Under hypoxic conditions, HIF‐1α up‐regulates p53 activation, increases miRNA‐34a, down‐regulates Klotho and promotes EMT in RPE cells, facilitating subretinal fibrosis and the progression of CNV. Moreover, blockade of the HIF‐1α/p53/miRNA‐34a axis alleviates subretinal fibrosis and the formation of CNV

Finally, we explored the effect of the HIF‐1α/p53/miRNA‐34a axis on subretinal fibrosis in a mouse laser‐induced CNV model. The protein levels of the CNV‐induced mesenchymal cell markers Fib, N‐cad and Vim were down‐regulated by HIF‐1α suppression, p53 mutation, miRNA‐34a inhibition and KL OE (Figure 6A‐D). Additionally, the CNV‐increased subretinal fibrosis area was relieved by HIF‐1α suppression, p53 mutation, miRNA‐34a inhibition and KL OE (Figure 6E,G). Moreover, HIF‐1α suppression, p53 mutation, miRNA‐34a inhibition and KL OE also reduced the volume (Figure 5E,F) of CNV. The data suggested that blockade of the HIF‐1α/p53/miRNA‐34a axis mitigated subretinal fibrosis and decreased CNV volume in a mouse laser‐induced CNV model.

## 4 DISCUSSION

Evidence suggests that the dysfunction of RPE cells precedes both the wet and dry types of AMD.^31^ As a monolayer of polarized cells, the RPE is essential for retinal homeostasis via multiple mechanisms, such as maintaining the outer blood‐retinal barrier (BRB), regulating nutrient and oxygen transportation to the outer retina and removing metabolic waste products from photoreceptors. However, under hypoxic conditions, RPE cells secrete excessive VEGF to exacerbate CNV.^32^ Moreover, subretinal fibrosis has recently been deemed a critical pathological event during CNV. Subretinal fibrosis is a process of fibrovascular proliferation consisting of vascular and fibrous components, the latter of which seems to be resistant to anti‐VEGF treatment. Continuous damage to the RPE and the outer layers of the neuronal retina in wet AMD increases the risk of subretinal fibrosis. RPE cells can undergo EMT during CNV to cause subretinal fibrosis.^2^ This study aimed to explore the molecular mechanism of RPE cell EMT during CNV.

In a CNV model induced by laser treatment in mice, the knockdown of HIF‐1α in RPE cells decreased the overexpression of VEGF and intercellular adhesion molecule 1 (ICAM‐1), reducing vascular leakage and CNV area,^33^ indicating that HIF‐1α derived from RPE cells promotes the progression of CNV. In this study, HIF‐1α produced by ARPE‐19 cells under hypoxic conditions was higher than that produced by normoxia‐challenged cells. In addition to inducing the transcription of VEGF and ICAM‐1, HIF‐1α also promotes fibrosis. For example, HIF‐1α is crucial for transforming growth factor β2 (TGF‐β2)‐induced EMT of human lens epithelial cells.^34^ Intriguingly, we found that HIF‐1α promoted ARPE‐19 cell EMT under hypoxic conditions.

The HIF‐1α target gene p53 also promotes fibrosis by enhancing the expression of its downstream target miRNA‐34a. In our study, the miRNA‐34a inhibitor alleviated CNV leakage, area and volume as well as subretinal fibrosis in mice indicating a potential therapy for the treatment of wet AMD. Moreover, intravenous administration of a miRNA‐34a inhibitor has been shown to ameliorate diabetes‐associated vascular endothelial dysfunction by up‐regulating Notch receptor 1 (NOTCH1).^35^ However, another study revealed that up‐regulation of miRNA‐34a gene expression partly inhibits the proliferation, migration and adhesion capabilities of ARPE‐19 cells by suppressing the expression of leucine‐rich repeat‐containing G protein‐coupled receptor 4 (LGR4).^36^ The difference in these findings may be due to the different culture conditions of ARPE‐19 cells. In our study, ARPE‐19 cells were exposed to hypoxic conditions, while ARPE‐19 cells were cultured under normoxic conditions in the other study.

Klotho is a membrane‐binding protein expressed in RPE cells.^21^ In its shed form, Klotho exerts anti‐fibrotic effects in several tissues, such as the kidney^37^ and heart.^38^ Furthermore, the absence of Klotho in mice accelerates ageing or progeroid syndromes and dramatically shortens the lifespan.^39^ In this study, we also found that Klotho levels decreased in hypoxia‐exposed ARPE‐19 cells and CNV lesions in mice compared with their normal controls.

In summary, our study demonstrates that the hypoxia‐induced HIF‐1α/p53/miRNA‐34a/Klotho axis in RPE cells promotes subretinal fibrosis and CNV formation. Several limitations of our study should be noted, such as the lack of primary RPE cell culture and the absence of gene editing in the mouse laser‐induced CNV model. Further studies are required to fully clarify the regulatory role of Klotho in RPE cell function during CNV.

## CONFLICT OF INTEREST

All authors declare that they have no conflicts of interest.

## AUTHOR CONTRIBUTIONS

**Laiqing Xie:** Conceptualization (equal); Investigation (equal); Writing‐original draft (equal). **Ying Wang:** Conceptualization (equal); Formal analysis (equal); Investigation (equal); Resources (equal); Supervision (equal). **Quan Li:** Data curation (equal); Formal analysis (equal); Investigation (equal); Methodology (equal); Resources (equal); Software (equal). **Xiaoyan Ji:** Conceptualization (equal); Investigation (equal); Resources (equal); Validation (equal). **Yuanyuan Tu:** Conceptualization (equal); Data curation (equal); Resources (equal); Software (equal); Supervision (equal). **Shu Du:** Conceptualization (equal); Investigation (equal); Visualization (equal). **Hui Lou:** Investigation (equal); Resources (equal). **Xinwei Zeng:** Investigation (equal); Resources (equal). **Linling Zhu:** Investigation (equal); Methodology (equal); Resources (equal). **Ji Zhang:** Conceptualization (equal); Data curation (equal); Funding acquisition (equal); Project administration (equal); Software (equal); Supervision (equal); Writing‐review & editing (equal). **Manhui Zhu:** Conceptualization (equal); Funding acquisition (equal); Project administration (equal); Writing‐review & editing (equal).

## Data Availability

The data that support the findings of this study are available from the corresponding author upon reasonable request.
